# Supplementary material for: Tlalpan 2020 Case Study: Enhancing Uric Acid Level Prediction with Machine Learning Regression and Cross-Feature Selection
Source: Nutrients. 2025 Mar 17;17(6):1052. doi: 10.3390/nu17061052 (PMC11946391; doi:10.3390/nu17061052)
Supplement: Supplementary file 1 [file nutrients-17-01052-s001.zip › nutrients-3499305-supplementary.pdf]

Article

Tlalpan 2020 Case Study: Enhancing Uric Acid Level Prediction with Machine Learning Regression and Cross-Feature Selection (Supplementary Materials)

Guadalupe Gutiérrez-Esparza [1,2,†,*], Mireya Martínez-García [3,†], Manlio F. Márquez-Murillo [2], Malinalli Brianza-Padilla [3], Enrique Hernández-Lemus [4,5*], Luis M. Amezcua-Guerra [3*]

[1] Researcher in the "Researcher for Mexico" Program under SECIHTI, Secretariat of Sciences, Humanities, Technology, and Innovation. Mexico City, 08400, Mexico
[2] Deputy Direction of Diagnostic and Treatment Services, National Institute of Cardiology Ignacio Chávez, Ciudad de México, México
[3] Department of Immunology, National Institute of Cardiology Ignacio Chávez, Ciudad de México, México
[4] Computational Genomics Division, National Institute of Genomic Medicine, Mexico City, Mexico
[5] Center for Complexity Sciences, Universidad Nacional Autónoma de México, México City, México
† Co-first authors
* Correspondence: ggutierreze@conahcyt.mx, ehernandez@inmegen.gob.mx, lmamezcuag@gmail.com

Version March 3, 2025 submitted to Nutrients

Table S1. Dataset variables

Name variable	Description	Type	Category
PhD	Doctorate	D	Academic level
Sec	high school	D	Academic level
Mstrs	Master's degree	D	Academic level
Bach	Preparatory school	D	Academic level
Prim	Primary education	D	Academic level
Prof	Professional	D	Academic level
Studnt	Student	D	Academic level
Tech	Technical	D	Academic level
Beer	Alcoholic beverage	D	Alcoholism data
Blueshot	Energy drink	D	Alcoholism data
Bomba	Energy drink	D	Alcoholism data
Boost	Energy drink	D	Alcoholism data
Brandy	Alcoholic beverage	D	Alcoholism data
Burn	Energy drink	D	Alcoholism data
Cognac	Alcoholic beverage	D	Alcoholism data
Liq	Liquors	D	Alcoholism data
Monster	Monster	D	Alcoholism data
Pulque	Alcoholic beverage	D	Alcoholism data
RedBul	Energy drink	D	Alcoholism data
RedWin	Alcoholic beverage	D	Alcoholism data
Redline	Energy drink	D	Alcoholism data
Rum	Rum	D	Alcoholism data
Shotb	Alcoholic beverage	D	Alcoholism data
Tequil	Alcoholic beverage	D	Alcoholism data

Table S1. Dataset variables

Name variable	Description	Type	Category
Venom	Energy drink	D	Alcoholism data
Vive100	Energy drink	D	Alcoholism data
Vodka	Alcoholic beverage	D	Alcoholism data
Whisky	Alcoholic beverage	D	Alcoholism data
WhiWin	Alcoholic beverage	D	Alcoholism data
AGE	Age	C	Anthropometric and Vital Data
Amput	Amputated	D	Anthropometric and Vital Data
AntInf	Anti-inflammatory drugs	D	Anthropometric and Vital Data
AntHTN	Antihypertensives	D	Anthropometric and Vital Data
AHR	Average Heart Rate	C	Anthropometric and Vital Data
BMI	Body Mass Index	D	Anthropometric and Vital Data
DPT1	Diastolic Pressure T1	C	Anthropometric and Vital Data
DPT2	Diastolic Pressure T2	C	Anthropometric and Vital Data
DPT3	Diastolic Pressure T3	C	Anthropometric and Vital Data
HRT1	Heart Rate T1	C	Anthropometric and Vital Data
HRT2	Heart Rate T2	C	Anthropometric and Vital Data
HRT3	Heart Rate T3	C	Anthropometric and Vital Data
HT	Height	C	Anthropometric and Vital Data
MAP	Mean Arterial Pressure	C	Anthropometric and Vital Data
DBP	Mean Diastolic Pressure	C	Anthropometric and Vital Data
SBP	Mean Systolic Pressure	C	Anthropometric and Vital Data
MI	Myocardial Infarction indicator	D	Anthropometric and Vital Data
PP	Pulse Pressure	C	Anthropometric and Vital Data
RR	Respiratory rate	D	Anthropometric and Vital Data
SPT1	Systolic Pressure T1	C	Anthropometric and Vital Data
SPT2	Systolic Pressure T2	C	Anthropometric and Vital Data
SPT3	Systolic Pressure T3	C	Anthropometric and Vital Data
UrTrct	Urinary tract	D	Anthropometric and Vital Data
WC	Waist circumference	C	Anthropometric and Vital Data
WGT	Weight	C	Anthropometric and Vital Data
IAT	Atherogenic Index	C	Blood and Urine Test Results
CHOLT	Total cholesterol	C	Blood and Urine Test Results
CREA	Creatinine	C	Blood and Urine Test Results
GLU	Glucose	C	Blood and Urine Test Results
HDLCO	HDL-Cholesterol	C	Blood and Urine Test Results
FE	Iron	C	Blood and Urine Test Results
LDLCO	LDL-Cholesterol	C	Blood and Urine Test Results
AntHLip	Lipid-lowering drugs	D	Blood and Urine Test Results
NA	Sodium	C	Blood and Urine Test Results
TRIG	Triglycerides	C	Blood and Urine Test Results
URIC	Uric Acid	C	Blood and Urine Test Results
CREO	Urine Creatinine	C	Blood and Urine Test Results
KO	Urine Potassium	C	Blood and Urine Test Results
NAO	Urine Sodium	C	Blood and Urine Test Results
Drunk	Drunkenness	D	Consumes alcoholic beverages
EnrgDrk	Energy drinks	D	Consumes alcoholic beverages
FreqDly	Frequency of alcohol ingestion: daily frequency	D	Consumes alcoholic beverages

Table S1. Dataset variables

Name variable	Description	Type	Category
Freq15d	Frequency of alcohol ingestion: every 15 days	D	Consumes alcoholic beverages
Freq3d	Frequency of alcohol ingestion: every third day	D	Consumes alcoholic beverages
FreqM1	Frequency of alcohol ingestion: once a month	D	Consumes alcoholic beverages
FreqWkE	Frequency of alcohol ingestion: weekends	D	Consumes alcoholic beverages
Gladiator	Energy drink	D	Consumes alcoholic beverages
Alcohol	Ingests	D	Consumes alcoholic beverages
MAmpu	Maternal amputation	D	Family medical history
MDiab	Mother with diabetes	D	Family medical history
MDysl	Mother with dyslipidemia	D	Family medical history
MEmbo	Maternal embolism	D	Family medical history
MGout	Maternal gout	D	Family medical history
MGFAm	Maternal grandfather amputation	D	Family medical history
MGFDi	Maternal grandfather diabetes	D	Family medical history
MGFDy	Maternal grandfather dyslipidemia	D	Family medical history
MGFEm	Maternal grandfather embolism	D	Family medical history
MGFGo	Maternal grandfather gout	D	Family medical history
MGFHA	Maternal grandfather heart attack	D	Family medical history
MGFHp	Maternal grandfather hypertension	D	Family medical history
MGFob	Maternal grandfather obesity	D	Family medical history
MGFSm	Maternal grandfather smoking	D	Family medical history
MGMAm	Maternal grandmother amputation	D	Family medical history
MGMDi	Maternal grandmother with diabetes	D	Family medical history
MGMDy	Maternal grandmother with dyslipidemia	D	Family medical history
MGMEm	Maternal grandmother with embolism	D	Family medical history
MGMGo	Maternal grandmother with gout	D	Family medical history
MGMHA	Maternal grandmother heart attack	D	Family medical history
MGMHp	Maternal grandmother with hypertension	D	Family medical history
MGMob	Maternal grandmother with obesity	D	Family medical history
MGMSm	Maternal grandmother smoking	D	Family medical history
MHAtt	Maternal heart attack	D	Family medical history
MHype	Mother with hypertension	D	Family medical history
Msobs	Maternal obesity	D	Family medical history
MSmok	Maternal smoking	D	Family medical history
PAmpu	Paternal amputation	D	Family medical history
PDiab	Paternal diabetes	D	Family medical history
PDysl	Paternal dyslipidemia	D	Family medical history
PEmbo	Paternal embolism	D	Family medical history
PGout	Paternal gout	D	Family medical history
PGFAm	Paternal grandfather amputation	D	Family medical history
PGFDi	Paternal grandfather diabetes	D	Family medical history
PGFDy	Paternal grandfather dyslipidemia	D	Family medical history
PGFEm	Paternal grandfather embolism	D	Family medical history
PGFGo	Paternal grandfather gout	D	Family medical history
PGFHA	Paternal grandfather heart attack	D	Family medical history
PGFHp	Paternal grandfather hypertension	D	Family medical history
PGFob	Paternal grandfather obesity	D	Family medical history
PGFSm	Paternal grandfather smoking	D	Family medical history

Table S1. Dataset variables

Name variable	Description	Type	Category
PGMAm	Paternal grandmother amputation	D	Family medical history
PGMDi	Paternal grandmother diabetes	D	Family medical history
PGMDy	Paternal grandmother dyslipidemia	D	Family medical history
PGMEm	Paternal grandmother embolism	D	Family medical history
PGMGo	Paternal grandmother gout	D	Family medical history
PGMHA	Paternal grandmother heart attack	D	Family medical history
PGMHp	Paternal grandmother hypertension	D	Family medical history
PGMob	Paternal grandmother obesity	D	Family medical history
PGMSm	Paternal grandmother smoking	D	Family medical history
PHAtt	Paternal heart attack	D	Family medical history
PHype	Father with hypertension	D	Family medical history
Psobs	Father with obesity	D	Family medical history
PSmok	Paternal smoking	D	Family medical history
CholBd	Bad cholesterol	D	General Health
Chol	Cholesterol	D	General Health
Diet	Diet	D	General Health
Embol	Embolism	D	General Health
CholGd	Good cholesterol	D	General Health
HiChol	High cholesterol	D	General Health
AntHGly	Hypoglycemics	D	General Health
Rehab	Rehabilitation	D	General Health
Kidney	Renal issues	D	General Health
Renal	Renal issues	D	General Health
ABRT	Abortions	D	Gyneco-obstetric data
BB4K	Baby > 4kg	D	Gyneco-obstetric data
CHB	Childbirths	D	Gyneco-obstetric data
CMPL	Complications in childbirth	D	Gyneco-obstetric data
CNTR	Contraceptives	D	Gyneco-obstetric data
MNP	Menopause	D	Gyneco-obstetric data
MNCL	Menstrual cycle	D	Gyneco-obstetric data
MENS	Menstruation	D	Gyneco-obstetric data
PRG	Pregnancies	D	Gyneco-obstetric data
EDM	Pregnancy edema	D	Gyneco-obstetric data
HYP	Pregnancy hypertension	D	Gyneco-obstetric data
PRECL	Pregnancy pre-eclampsia	D	Gyneco-obstetric data
SGR	Pregnancy sugar	D	Gyneco-obstetric data
ELCT	Electric vehicle	D	Mobility and Transport
MTR	Motor vehicle	D	Mobility and Transport
PRC	Particular vehicle	D	Mobility and Transport
TXI	Taxi	D	Mobility and Transport
Driver	Driver	D	Occupation
HouseW	Housewife	D	Occupation
Merch	Merchant	D	Occupation
Profn	Professional worker	D	Occupation
Retire	Retired	D	Occupation
WrkHbl	Skilled worker	D	Occupation
Study	Studying	D	Occupation

Table S1. Dataset variables

Name variable	Description	Type	Category
Unemp	Unemployed	D	Occupation
BCL	Bicycle	D	Physical activity data
High	High physical activity	D	Physical activity data
Low	Low physical activity	D	Physical activity data
Med	Medium physical activity	D	Physical activity data
ActPhy	Physical activity	D	Physical activity data
VLow	Very low physical activity	D	Physical activity data
WLK	Walking	D	Physical activity data
ASH	Average Sleep Hours	C	Sleep quality
DTN	Daytime Naps	C	Sleep quality
DTS	Daytime Sleepiness	C	Sleep quality
Dx	Sleep apnea diagnosis	D	Sleep quality
Drowsy	Drowsiness	C	Sleep quality
ENGS	Enough Sleep	C	Sleep quality
Fatig	Fatigue	C	Sleep quality
FRST	Feel Rested	C	Sleep quality
RLS	Restless Sleep	C	Sleep quality
SLPA2	sleep adequacy	C	Sleep quality
SLPD4	sleep disturbance	C	Sleep quality
SLP6	sleep problems index I	C	Sleep quality
SLP9	sleep problems index II	C	Sleep quality
SLPSOB1	sleep short of breath or headache	C	Sleep quality
SLPS3	SLPS3 sleep somnolence	C	Sleep quality
SleepQ	Snore	C	Sleep quality
SLPSNR1	snoring	C	Sleep quality
TTS	Time to Sleep	C	Sleep quality
TSLP	Trouble Sleeping	C	Sleep quality
TSA	Trouble Staying Awake	C	Sleep quality
TWU	Trouble Waking Up	C	Sleep quality
WBH	Wake with Breathlessness or Headache	C	Sleep quality
SmkNow	Currently smokes	D	Smoking Status Data
SmkDay	Daily smoker	D	Smoking Status Data
ExSmk	Ex-smoker	D	Smoking Status Data
SmkOcc	Occasional smoker	D	Smoking Status Data
SmkPas	Passive smoker	D	Smoking Status Data
Smoke	Smoked	D	Smoking Status Data
BiPar	Biparental family	D	Socioeconomic and Family Data
MonoPar	Monoparental family	D	Socioeconomic and Family Data
Recon	Reconstructed family	D	Socioeconomic and Family Data
Salary22_44	Salary range 2, 200.00*to*4,400.00	D	Socioeconomic and Family Data
Salary44_66	Salary range 4, 400.00*to*6,600.00	D	Socioeconomic and Family Data
Salary66_110	Salary range 6, 600.00*to*11,000.00	D	Socioeconomic and Family Data
SalaryTo22	Salary range up to $2,200.00	D	Socioeconomic and Family Data
Salary110	The salary range of over $11,000.00	D	Socioeconomic and Family Data
ISSSTE	Institute for Social Security and Services for State Workers	D	Types of Medical Insurance
SvcMed	Medical service	D	Types of Medical Insurance

Table S1. Dataset variables

Name variable	Description	Type	Category
IMSS	Mexican Social Security Institute	D	Types of Medical Insurance
SegPop	Popular Insurance	D	Types of Medical Insurance
Priv	Private insurance	D	Types of Medical Insurance

© 2025 by the authors. Submitted to *Nutrients* for possible open access publication under the terms and conditions of the Creative Commons Attribution (CC BY) license (http://creativecommons.org/licenses/by/4.0/).

Table S2. Performance metrics of the Cross-Feature Selection method for men and women

Women					Men				
**Max_iter**	**Max_depth**	**l2_regularization**	**Mean_r2**	**Std_r2**	**Max_iter**	**Max_depth**	**l2_regularization**	**Mean_r2**	**Std_r2**
50	3	0	0.2649	0.0410	50	3	0	0.1712	0.0296
50	3	0.1	0.2612	0.0409	50	3	0.1	0.1741	0.0340
50	3	0.5	0.2649	0.0411	50	3	0.5	0.1712	0.0344
50	5	0	0.2514	0.0554	50	5	0	0.1509	0.0380
50	5	0.1	0.2503	0.0528	50	5	0.1	0.1495	0.0470
50	5	0.5	0.2492	0.0538	50	5	0.5	0.1585	0.0407
50	7	0	0.2285	0.0512	50	7	0	0.1299	0.0574
50	7	0.1	0.2238	0.0512	50	7	0.1	0.1345	0.0458
50	7	0.5	0.2284	0.0591	50	7	0.5	0.1404	0.0484
100	3	0	0.2568	0.0575	100	3	0	0.1593	0.0346
100	3	0.1	0.2510	0.0545	100	3	0.1	0.1679	0.0429
100	3	0.5	0.2582	0.0584	100	3	0.5	0.1617	0.0411
100	5	0	0.2366	0.0636	100	5	0	0.1229	0.0422
100	5	0.1	0.2300	0.0593	100	5	0.1	0.1194	0.0513
100	5	0.5	0.2295	0.0674	100	5	0.5	0.1253	0.0475
100	7	0	0.2036	0.0643	100	7	0	0.0870	0.0579
100	7	0.1	0.2035	0.0636	100	7	0.1	0.0989	0.0497
100	7	0.5	0.2068	0.0706	100	7	0.5	0.1031	0.0526
150	3	0	0.2451	0.0578	150	3	0	0.1430	0.0451
150	3	0.1	0.2401	0.0567	150	3	0.1	0.1488	0.0479
150	3	0.5	0.2452	0.0626	150	3	0.5	0.1501	0.0467
150	5	0	0.2201	0.0693	150	5	0	0.0962	0.0449
150	5	0.1	0.2137	0.0679	150	5	0.1	0.0953	0.0587
150	5	0.5	0.2105	0.0714	150	5	0.5	0.1020	0.0488
150	7	0	0.1889	0.0704	150	7	0	0.0623	0.0613
150	7	0.1	0.1892	0.0693	150	7	0.1	0.0730	0.0521
150	7	0.5	0.1850	0.0773	150	7	0.5	0.0783	0.0615
